# Supplementary material for: Sphingosine Kinase 1/S1P Signaling Contributes to Pulmonary Fibrosis by Activating Hippo/YAP Pathway and Mitochondrial Reactive Oxygen Species in Lung Fibroblasts
Source: Int J Mol Sci. 2020 Mar 17;21(6):2064. doi: 10.3390/ijms21062064 (PMC7139883; doi:10.3390/ijms21062064)
Supplement: Supplementary file 1 [file ijms-21-02064-s001.pdf]

International Journal of *Molecular Sciences*

MDPI

Supplementary data

Sphingosine kinase 1/S1P signaling contributes to pulmonary fibrosis by activating Hippo/YAP pathway and mitochondrial reactive oxygen species in lung fibroblasts

Long Shuang Huang[1#], Tara Sudhadevi[2#], Panfeng Fu[1], Prasanth-Kumar Punathil-Kannan[1], David L. Ebenezer[1], Ramaswamy Ramchandran[1], Vijay Putherickal[1], Paul Cheresh[3,4], Guofei Zhou[2], Alison W. Ha[5], Anantha Harijith[2], David W. Kamp[3,4] and Viswanathan Natarajan[1,6] *

Departments of [1]Pharmacology, [2]Pediatrics, [5]Biochemistry and Molecular genetics, and [6]Medicine, University of Illinois at Chicago, Chicago, Illinois, USA; [3]Department of Medicine, Division of Pulmonary & Critical care Medicine, Jesse Brown VA Medical Center, Chicago, Illinois, USA; [4]Department of Medicine, Northwestern University Feinberg School of Medicine, Chicago, IL, USA.

***** Corresponding Author: Viswanathan Natarajan, PhD, Department of Pharmacology, Room #3137, COMRB Building, 909, South Wolcott Avenue, Chicago, IL 60612; Tel: 312-355-5896, Fax: 312-996-7193; Email: visnatar@uic.edu

# contributed equally to the manuscript

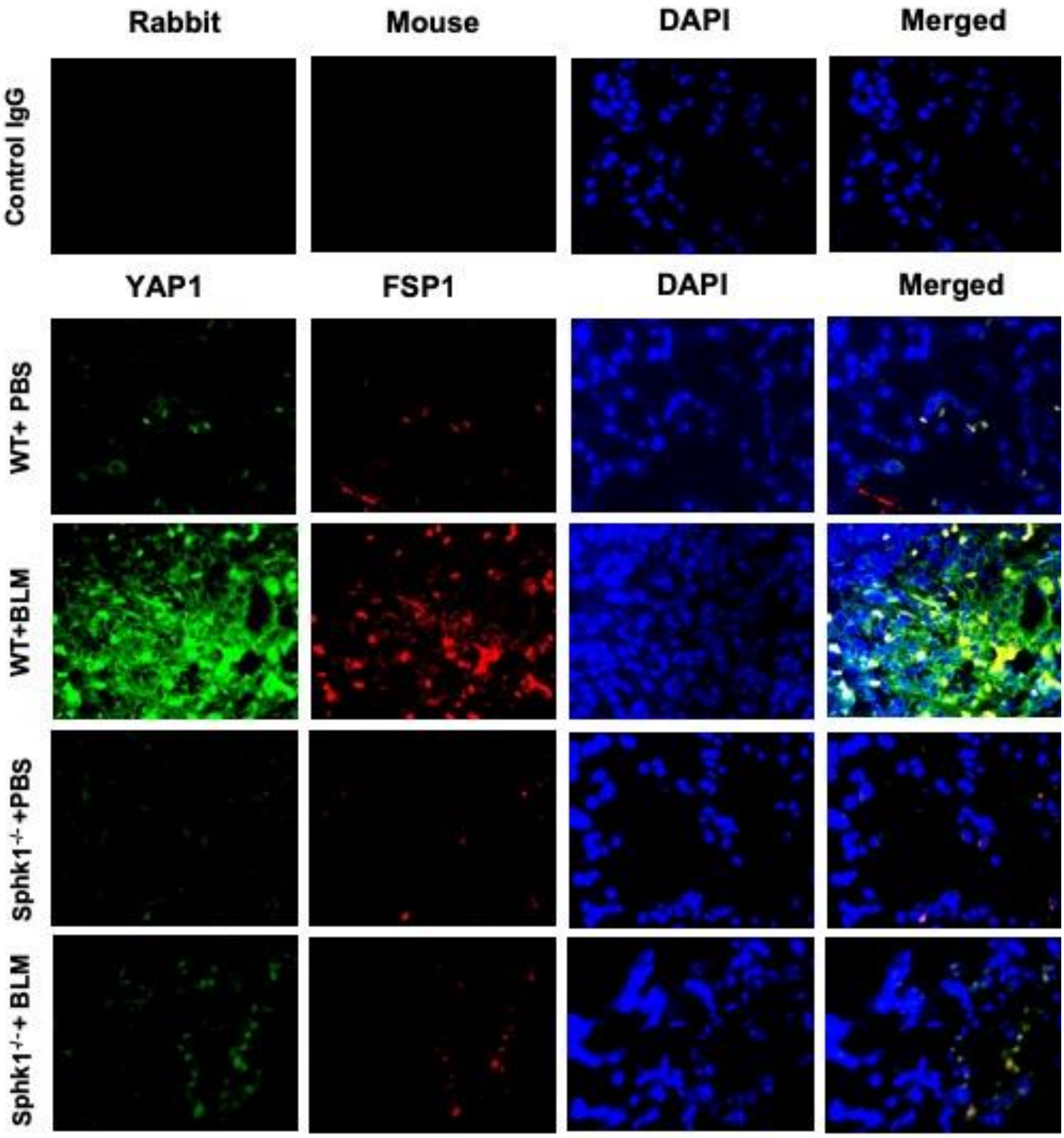

Supplementary Fig. 1. Genetic deletion of *Sphk1* in fibroblasts reduces bleomycin- and TGF-β-induced YAP1 expression. (**A & B**) *Sphk1$^{flox/flox}$* and *Sphk1$^{flox/flox}$: FSP1Cre^{+}* mice (male, 8 weeks) in C57BL/6 background receiving bleomycin (2 U/kg in 50 µl PBS) or PBS intratracheally were sacrificed at days 21 post-challenge. The conditional deletion of *Sphk1* in fibroblasts reduced bleomycin-induced YAP expression (green) and FSP1 expression (red) and their colocalization as seen with the merged image (yellow). Row1 represents the IgG control (isotype) for the specific antibodies. Row 2 represents the YAP1 (green), FSP1 (red), DAPI (blue) and the merged images (yellow to orange) for the wild type mice injected with PBS. Row 3 represents the YAP1 (green), FSP1 (red), DAPI (blue) and the merged images (yellow) for the wild type mice administered with bleomycin, where maximum colocalization of YAP1 and FSP could be seen. Row 4 represents the YAP1 (green), FSP1 (red), DAPI (blue) and the merged images (yellow to orange) for the *Sphk1$^{-/-}$* mice administered with PBS. The last row (row 5) represents the YAP1 (green), FSP1 (red), DAPI (blue) and the merged images (yellow) for the *Sphk1$^{-/-}$* mice administered with bleomycin, where the colocalization is much reduced. n=4 to 6 per group.

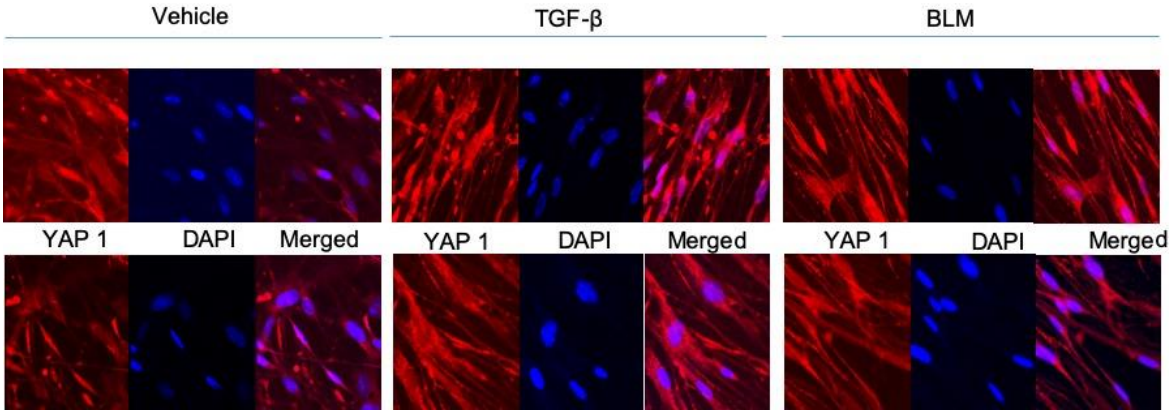

Supplementary Fig. 2. PF543 attenuates TGF-β- and bleomycin-mediated YAP1 translocation to cell nucleus. Human lung fibroblasts (HLFs) grown on glass-bottom 35-mm dishes were treated with PF543 (1 μM) for 1 h prior to TGF-β (5 ng/ml) or bleomycin (BLM) (1 U/ml) for 3 h, and YAP1 translocation to cell nucleus was assessed by confocal microscopy. The individual as well as merged images of the vehicle as well as TGF-β and bleomycin induced cells are shown in the panel. Upon treatment with bleomycin or TGF-β, YAP-1 translocation to nucleus is seen (pink) which is reverted with PF543 treatment (less pink and more of blue). Seven different areas from three independent dishes were counted for quantification of nuclear YAP1 after treatment vs. control without treatment. YAP1 is shown in red, and DAPI in blue and translocation is identified by pink nucleus. n=3.

Figure 1	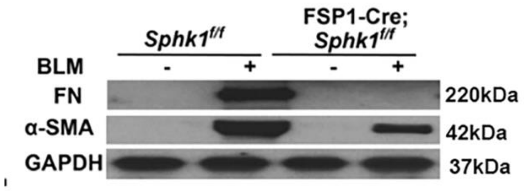
FN	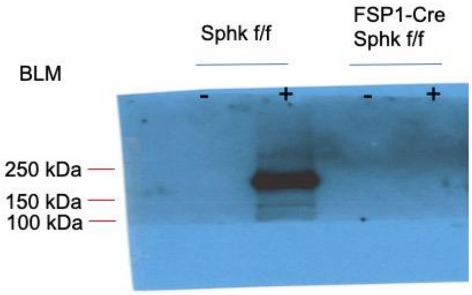
α -SMA	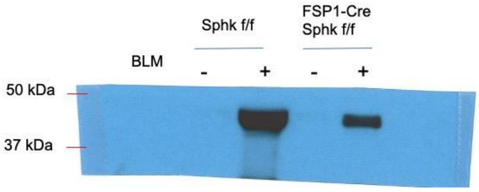
GAPDH	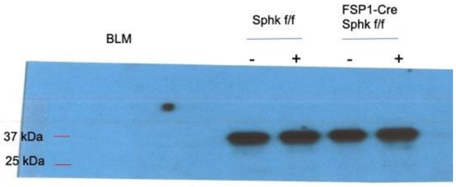

Supplementary Fig. 3. (original blots of Fig. 1G.)

Figure 2

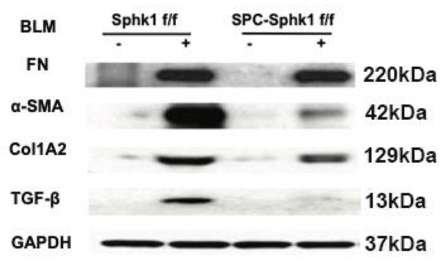

FN

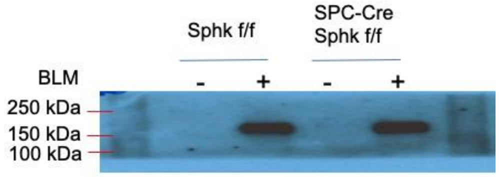

α -SMA

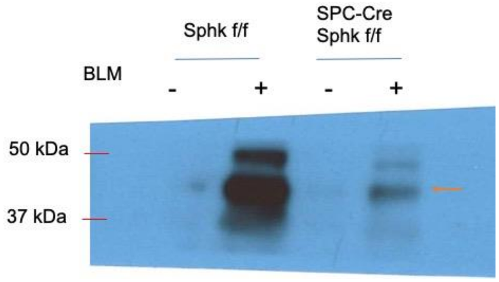

Col 1A2

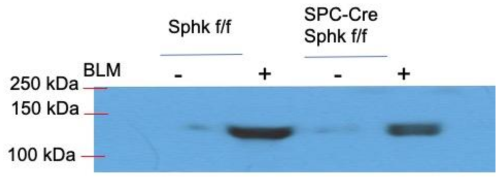

TGF-β

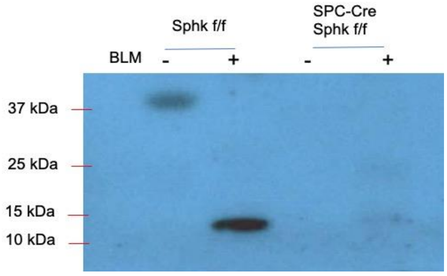

GAPDH

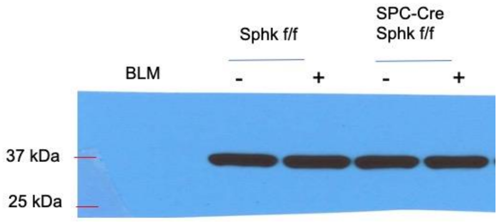

Supplementary Fig. 4. (original blots of Fig. 2C.)

Figure 4	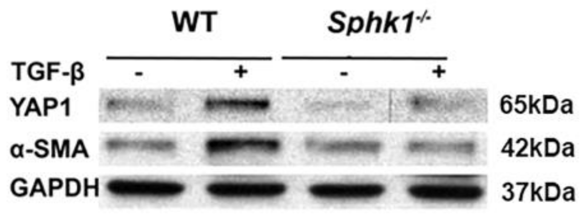
YAP1	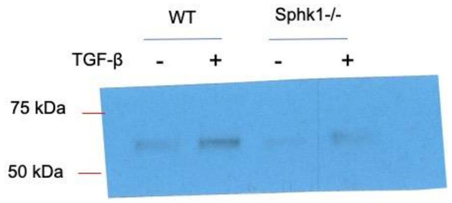
α -SMA	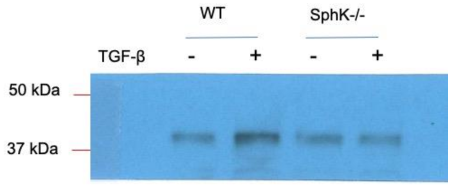
GAPDH	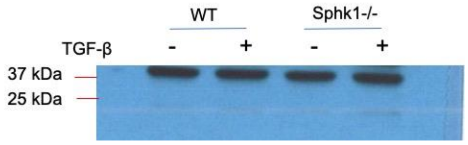

Supplementary Fig. 5. (original blots of Fig. 4C.)

<table>
<tr><td>Figure 5</td><td>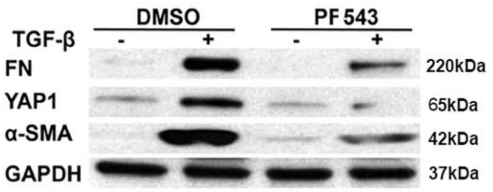
</td></tr>
<tr><td>FN</td><td>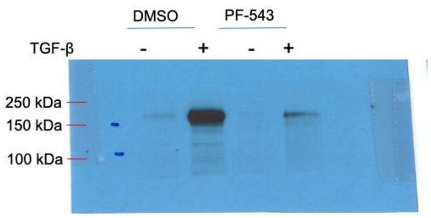
</td></tr>
<tr><td>YAP1</td><td>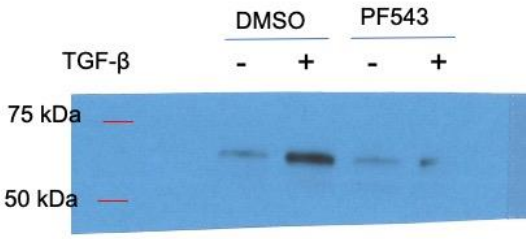
</td></tr>
<tr><td>α -SMA</td><td>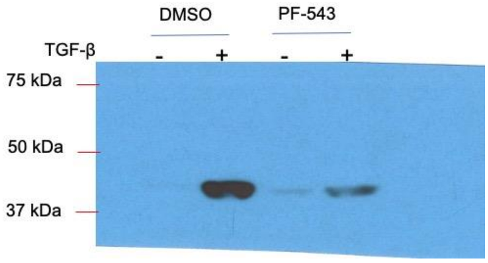
</td></tr>
<tr><td>GAPDH</td><td>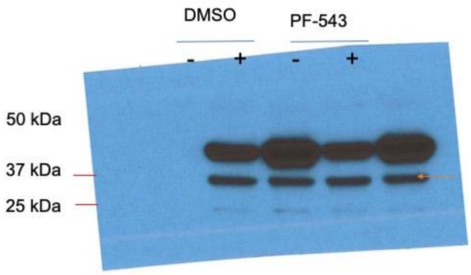
</td></tr>
</table>

Supplementary Fig. 6. (original blots of Fig. 5C.)

Figure 6

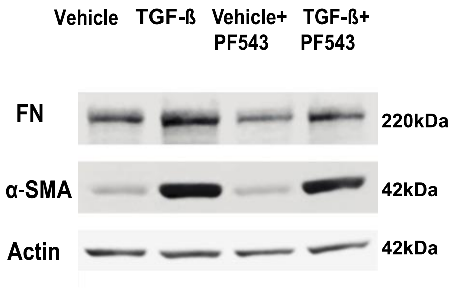

FN

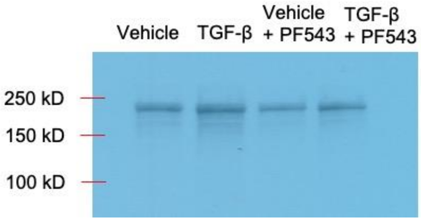

α -SMA

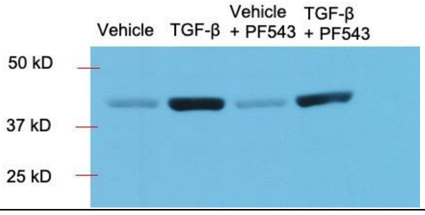

Actin

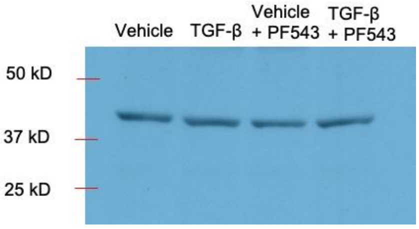

Supplementary Fig. 7. (original blots of Fig. 6A.)

Figure 8	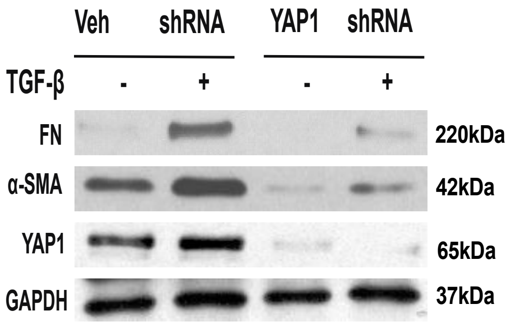
FN	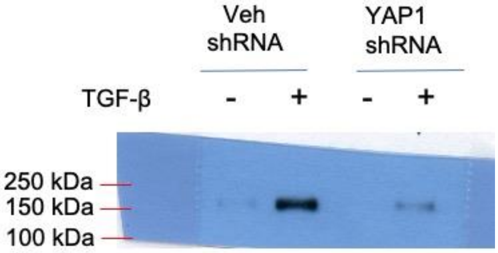
YAP1	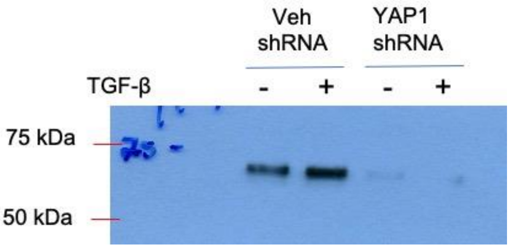
α -SMA	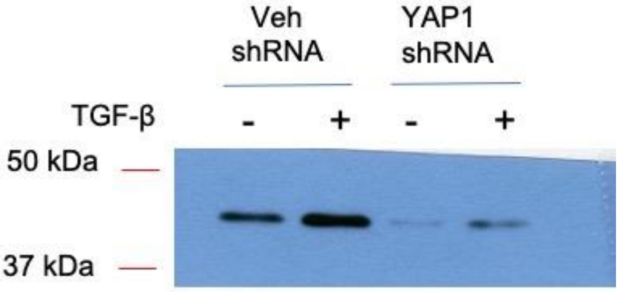
GAPDH	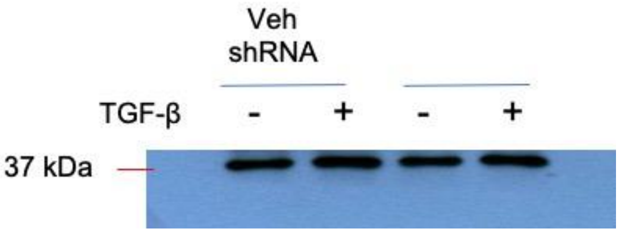

Supplementary Fig. 8. (original blots of Fig. 8B.)

Figure 9	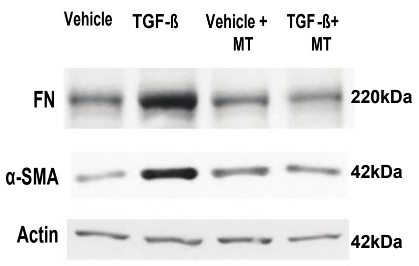
FN	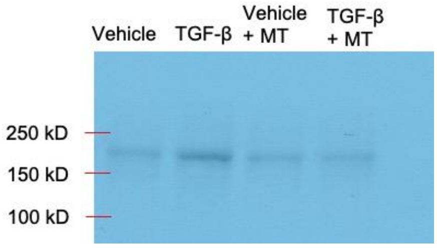
α -SMA	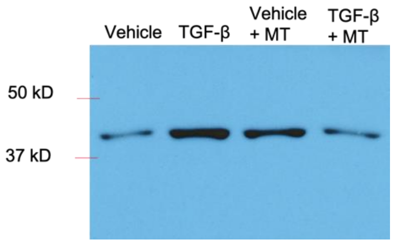
Actin	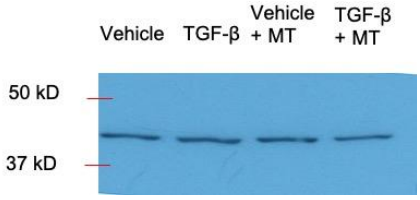

Supplementary Fig. 9. (original blots of Fig. 9B.)
